# Economic impact of clinical and subclinical mastitis: a herd-level cost analysis in dairy cattle farms of Lugo (Northwest of Spain)

**DOI:** 10.1093/tas/txag081

**Published:** 2026-06-18

**Authors:** J Álvarez, E N Martínez, M García, J Hernández, R Muiño, A Acción, R Barrionuevo, U Yáñez, A I Peña, J J Becerra, L A Quintela

**Affiliations:** Unit of Reproduction and obstetrics. Department of Animal Pathology. Faculty of veterinary, Campus Terra, Universidade de Santiago de Compostela. Avda. Carballo Calero s/n, Lugo, 27002, España; Departament of Animal Pathology, Campus Terra-IBADER, Universidade de Santiago de Compostela. Avda. Carballo Calero s/n, Lugo, 27002, España; Meira Veterinary Center, Galicia, Spain; Departament of Animal Pathology, Campus Terra-IBADER, Universidade de Santiago de Compostela. Avda. Carballo Calero s/n, Lugo, 27002, España; Departament of Animal Pathology, Campus Terra-IBADER, Universidade de Santiago de Compostela. Avda. Carballo Calero s/n, Lugo, 27002, España; Unit of Reproduction and obstetrics. Department of Animal Pathology. Faculty of veterinary, Campus Terra, Universidade de Santiago de Compostela. Avda. Carballo Calero s/n, Lugo, 27002, España; Unit of Reproduction and obstetrics. Department of Animal Pathology. Faculty of veterinary, Campus Terra, Universidade de Santiago de Compostela. Avda. Carballo Calero s/n, Lugo, 27002, España; Unit of Reproduction and obstetrics. Department of Animal Pathology. Faculty of veterinary, Campus Terra, Universidade de Santiago de Compostela. Avda. Carballo Calero s/n, Lugo, 27002, España; Unit of Reproduction and obstetrics. Department of Animal Pathology. Faculty of veterinary, Campus Terra, Universidade de Santiago de Compostela. Avda. Carballo Calero s/n, Lugo, 27002, España; Unit of Reproduction and obstetrics. Department of Animal Pathology. Faculty of veterinary, Campus Terra, Universidade de Santiago de Compostela. Avda. Carballo Calero s/n, Lugo, 27002, España; Departament of Animal Pathology, Campus Terra-IBADER, Universidade de Santiago de Compostela. Avda. Carballo Calero s/n, Lugo, 27002, España; Unit of Reproduction and obstetrics. Department of Animal Pathology. Faculty of veterinary, Campus Terra, Universidade de Santiago de Compostela. Avda. Carballo Calero s/n, Lugo, 27002, España; Departament of Animal Pathology, Campus Terra-IBADER, Universidade de Santiago de Compostela. Avda. Carballo Calero s/n, Lugo, 27002, España

**Keywords:** economic impact, cost analysis, milk production losses, mathematical modelling, farm profitability

## Abstract

Mastitis remains one of the most important causes of economic loss in dairy farms. However, few studies have quantified the herd-level costs of both clinical (CM) and subclinical (SM) mastitis. The objectives of this study were to estimate the annual economic impact of CM and SM in dairy farms located in Northwest Spain, to disaggregate costs into their principal direct and indirect components, and to assess the relative contribution of both presentations of mastitis to total economic losses. A retrospective observational herd-level economic analysis was conducted using production, health, reproductive, and economic data collected during 2023 from 63 commercial dairy farms enrolled in a milk recording and quality control program. A deterministic economic model integrating farm-specific data with coefficients derived from the literature was used to estimate direct and indirect costs. Parameter uncertainty was evaluated through a Monte Carlo simulation with 10,000 iterations and a dominance analysis. Average mastitis total cost was €31,643.48 per farm per year (€261.23/cow). Subclinical mastitis accounted for €18,480.56 (58.4%) and CM for €13,162.99 (41.6%). In the deterministic analysis, milk production losses were the largest contributor to both CM (€3679.60 ± 5954.92) and SM (€15,643.70 ± 14,797.20) costs. However, under uncertainty, culling-related costs were the most frequent primary driver of total economic losses (72.2% of simulations), followed by increased days open (21.9%) and milk-related losses (5.9%). Mastitis represents a substantial economic burden in Atlantic dairy systems. Although milk losses are consistently important, culling and reproductive inefficiency may dominate total costs under plausible biological and economic conditions.

## Teaser

Mastitis silently drains profits from dairy farms, costing an average of €31,600 per farm each year. Our study in Northwest Spain shows that subclinical cases, often hidden from view, are the costliest.

## Introduction

Mastitis is a multi-etiological disease characterized by inflammation of the mammary gland, most commonly caused by bacterial infection, and remains one of the most important health disorders affecting dairy cattle worldwide (Stanek et al. 2024). According to the presence or absence of clinical signs, mastitis is classified as clinical mastitis (CM) or subclinical mastitis (SM) (Peters et al. 2015; Nakada et al. 2023). Clinical mastitis is characterized by visible abnormalities in the milk and/or signs of udder inflammation, which may be accompanied by systemic illness (Peters et al. 2015). On the other hand, SM occurs in the absence of clinical signs and is detected through increased somatic cell counts; it is considered the most prevalent presentation of mastitis (Peters et al. 2015). Both CM and SM are among the most common diseases in dairies (Borş et al. 2023; Nakada et al. 2023; Miyata et al. 2024).

In addition to the substantial investments required for mastitis prevention and control, this disease generates considerable direct and indirect economic losses (Aghamohammadi et al. 2018). Over the past decades, multiple studies have estimated mastitis costs, reporting considerable variability among countries and production systems, with costs ranging from €117 to €1496 (Halasa et al. 2007; Pérez-Cabal et al. 2008; Aghamohammadi et al. 2018) (Heikkilä et al., 2012). This variability reflects differences in herd characteristics, milk production and price, labor costs, veterinary expenses, and replacement values, as well as regional and temporal fluctuations in economic conditions (Halasa et al. 2007; Aghamohammadi et al. 2018; Corrêa et al. 2024).

Economic losses associated with mastitis are classified as direct and indirect costs (Aghamohammadi et al. 2018). Direct costs are primarily associated with CM and include diagnostic procedures, veterinary services, antimicrobial and anti-inflammatory treatments, discarded milk, additional labor, and, in severe cases, mortality (Aghamohammadi et al. 2018; Corrêa et al. 2024). Indirect costs are mostly related to chronic mastitis and SM (Corrêa et al. 2024), and include reduced milk production, impaired reproductive performance, and increased risk of culling (Halasa et al. 2007; Heikkilä et al., 2012). It is estimated that only approximately 30% of mastitis-associated costs are easily detected by farmers, while indirect losses often account for most of the total economic burden (Corrêa et al. 2024).

Although the economic impact of mastitis has been widely studied, important knowledge gaps persist. Many previous studies have relied on simulation-based approaches, focused exclusively on clinical mastitis, or evaluated a limited number of cost components. In addition, relatively few studies are based on real farm-level data integrating production, reproductive performance, and detailed economic inputs, particularly in Southern European dairy systems under current milk price conditions. As a result, the relative contribution of clinical and subclinical mastitis to the total herd-level economic losses remains insufficiently characterized.

Therefore, the objectives of this study were (1) to estimate the annual herd-level economic costs of clinical and subclinical mastitis in dairy farms located in Lugo (Northwest Spain) using real production and economic data; (2) to quantify and disaggregate these costs into their main direct and indirect components through a structured herd-level economic calculation model; and (3) to assess the relative contribution of clinical and subclinical mastitis to total mastitis-associated costs under Atlantic European dairy farming conditions.

## Material and methods

### Study design and herd selection

This study was designed as an observational, retrospective herd-level economic analysis. Data were collected from 63 conventional commercial dairy farms distributed across the Terra Chá region (Lugo, Galicia, Spain), a lowland plain of approximately 1822 km^2^. Galicia, and particularly the province of Lugo, represents the core of intensive dairy production in Spain, accounting for the highest concentration of dairy farms (57%) and dairy cows (41%) in the country (Secretaría General Técnica 2025; IGE 2025). The region is characterized by professionalized, medium- to large-sized herds operating under Atlantic climatic conditions, with high annual rainfall and moderate temperatures (Otero Rodríguez and Lanero Táboas, 2023). These characteristics are comparable to those observed in other Atlantic European dairy regions such as northern Portugal, western France, and the Netherlands, where mastitis also remains one of the main health and economic challenges in dairy production. The study period covered 12 consecutive months (January–December 2023). All farms were independent operations, and cows were housed and managed separately at each individual facility.

The studied herds had an average size of 126.4 ± 99.32 dairy cows (mean ± *SD*), comprising a total of 8309 animals (5634 multiparous and 2675 primiparous) (Table 1). All participating farms were voluntarily enrolled in the Africor Lugo milk recording and quality control program, which provides standardized test-day records, including average milk production per cow (kg/cow/lactation; Table 1), average 305 days milk production (kg/cow/year), average lactation length, culling rate, death rate, average bulk tank somatic cells count (BTSCC), average dry period length (days) and days open. Only farms with complete production, health, and economic records for the study period were included in the analysis. Although the sample was not randomly selected, the participating herds represent professional dairy farms operations typical of intensive Atlantic production systems in Northwest Spain. However, potential selection bias associated with voluntary participation is acknowledged.

**Table 1:** Summary of herd characteristics and productive parameters for the 63 farms located in Northwest Spain included in the study.

	Mean	*SD*	Minimum	Maximum
***Multiparous (Cows)***	84.37	65.68	14.00	278.00
***Primiparous (Cows)***	42.00	34.74	4.00	141.00
***Total milking cows (Cows)***	126.37	99.32	22.00	419.00
***Average production 305 days (Kg)***	9432.04	1812.69	5438.40	12,534.20
***Average complete milk production (Kg)***	11,616.60	2201.55	6062.00	15,684.24
***Average lactation duration (Days)***	343.51	32.33	276.00	449.70
***% Fat***	3.90	0.20	3.43	4.32
***% Protein***	3.36	0.11	2.98	3.56
***Average milk production per day (Kg)***	33.90	6.10	18.63	43.10
***Total annual milk production (Kg)***	1,491,419.93	1,287,788.08	151,548.00	4,980,359.70
***Culling rate (%)***	31.29	10.26	5.76	69.17
***Death rate (%)***	3.38	3.31	0.00	13.04
***Somatic cells count***	200.37	59.82	79.07	434.40
***Average dry period (Days)***	58.74	9.55	32.20	85.00
***Average days open***	122.24	28.19	74.50	227.00
***Clinical mastitis (%)***	13.78	11.84	0.50	61.81
***Death due mastitis (%)***	1.33	2.02	0.00	8.20
***Culling cows due mastitis (%)***	6.01	6.21	0.38	26.30

### Definition and recording of clinical mastitis

Clinical mastitis was defined as the presence of visible abnormalities in the milk (such as clots, flakes, watery appearance, or blood) and/or inflammatory signs in the udder or affected quarter (including swelling, redness, heat, and pain), confirmed by a positive California Mastitis Test (CMT).

Cows were examined daily for the detection of clinical mastitis by routine visual inspection of the udder during milking. Milk samples were collected from individual quarters at the time of a clinical mastitis episode and submitted to the Galician Interprofessional Milk Analysis Laboratory (GIMAL) for bacteriological culture and antimicrobial susceptibility testing. Somatic cell count (SCC) was determined for each quarter-level milk sample. An SCC value above 400,000 cells/mL was considered indicative of infection. Laboratory results, including SCC, pathogen identification, and antibiogram, were reported simultaneously to both the farmer and the attending veterinarian. Depending on the severity of the case, the farmer either requests veterinary assistance or initiates treatment based on a pre-established protocol agreed with the veterinarian specialized in milk quality. All these herds were enrolled in a milk quality service, whereby a veterinarian conducted at least one visit per farm per month. In cases of increased incidence of subclinical or clinical mastitis, the frequency of herd visits increased accordingly.

All udder-health and economic variables were recorded at each visit. To address time variation across visits, data were subsequently aggregated at the herd level.

### Economic model structure

A deterministic herd-level economic calculation model was used to estimate the costs associated with clinical and subclinical mastitis. Total mastitis-associated cost was calculated as the sum of direct and indirect components. The model integrates farm-specific inputs (mastitis incidence, milk production, reproductive performance, and economic variables) with cost coefficients derived from peer-reviewed literature. Costs were calculated separately for clinical and subclinical mastitis and subsequently aggregated. The deterministic model provides point estimates of mastitis-related costs based on observed farm data and fixed parameter values. The percentage of clinical mastitis was calculated using cumulative records over the study period. Economic variables subjected to temporal variation, including milk price, veterinary services, diagnostic costs, and treatment expenses, were averaged across the monthly visits to obtain representative annual values for each farm.

### Parameterization of cost components

Cost coefficients and biological parameters (including milk yield reduction, additional days open, relative risk of culling and mortality) were obtained through a structured review. For the deterministic analysis, a single representative value (central estimate) was selected for each parameter based on the available evidence. Detailed calculations for each cost component, including diagnostic costs, treatment protocols, discarded milk, labor requirements, mortality, culling, and reproductive losses, were performed as summarized in Supplementary Table S1, available as supplementary data at *Translational Animal Science* online.

#### Clinical mastitis

DIRECT COSTS:

Diagnostic cost:

Treatment-related costs were recorded for all mastitis cases, regardless of whether treatment was administered by the farmer or by a veterinarian. When mastitis was diagnosed and treated by the farmer following prevention and treatment protocols previously established by a veterinarian, no veterinary visit cost was applied. Veterinary visit costs were included only in cases where a veterinarian attended the farm to diagnose or manage mastitis. On farms where no treatment protocols existed and a veterinarian was required for each mastitis case, the minimum professional time per visit was estimated at 30 minutes (Richert et al. 2013).

Cost of tests performed for diagnosis:

This includes expenses related to sending samples to a laboratory for culture to identify the microorganisms causing mastitis and for performing antibiotic susceptibility tests (antibiograms).

Treatment cost:

Every case of clinical mastitis was treated as soon as possible to minimize production losses. Treatment strategies were based on the clinical severity of mastitis. In cases presenting local signs only, such as udder inflammation and abnormal milk without general clinical symptoms, intramammary antimicrobial therapy was applied, with the addition of anti-inflammatory drugs when gland inflammation was evident. In cases presenting general clinical signs, a combined therapeutic approach was used, including intramammary antimicrobial therapy, anti-inflammatory treatment, and systemic antimicrobial therapy.

On each farm, the average cost of the treatments applied during the study period was recorded and included in the economic analysis.

Discarded milk cost:

This refers to the value of the milk that must be discarded during treatment. This cost depends on the number of treatment days, the withdrawal period of the administered medications, and the cow’s milk production at that time, making it highly variable.

Cost of deaths due to mastitis:

The risk of a cow dying from mastitis is relatively low (OR: 1.2) (Seegers et al. 2003), but the death of a single animal represents a significant cost to the farm. For this reason, the proportion of direct costs attributed to death is often higher than that of treatment. Using this data, and knowing the incidence of mastitis and the farm’s mortality rate, we calculated the percentage of deaths due to mastitis. The cost of a death is equivalent to the cost of replacing the animal.

Labor cost:

Every sick animal on a farm requires additional time beyond normal care, even if only for treatment administration. In the case of CM, the estimated time spent on a cow is around 45 minutes (throughout the process) (van Soest et al. 2016). While the worker or farmer is engaged in this task, they cannot perform other duties on the farm, so this time must be considered a cost. This cost will depend on the hourly labor cost on the farm.

INDIRECT COSTS:

Decrease in production:

The effect of CM on milk production varies depending on several factors, particularly the timing of the occurrence. The reduction can range from 8% when CM occurs in the first month of lactation, to 1% when it occurs in the ninth month. However, based on the distribution of clinical mastitis throughout lactation, we can estimate an average production loss of 5% per mastitis case (Huijps et al. 2008).

Increased culling:

The risk of culling a cow with mastitis is 1.8 times higher than for a healthy cow (Seegers et al. 2003). As with mortality, using this data and knowing the incidence of mastitis and the culling rate of the farm, we can calculate the percentage of culls due to mastitis. The cost of culling is the difference between the replacement cost and the residual value of the cow.

Reduced reproductive efficiency:

The negative effects that mastitis can have on reproduction include:

- Reduced fertility when mastitis occurs within 30 days before or after artificial insemination, with greater negative impact the closer it occurs to insemination (Nava-Trujillo et al. 2010).
- Pregnancy loss throughout gestation after the onset of CM. Specifically, studies have shown that cows with mastitis within the first month and a half of gestation are 2.5 times more likely to lose pregnancy in the first three months than cows without mastitis. (Nava-Trujillo et al. 2010)

Ultimately, these problems lead to an increase in the interval between calving and successful insemination, and thus a longer calving interval. The magnitude of this increase varies between 10 and 68 days depending on the study (Santos et al. 2004; Ahmadzadeh et al. 2009; Nava-Trujillo et al. 2010; Nava-Trujillo, 2019). For our calculations, we used 20 days as a conservative estimate:

- Calculation: Increase of 20 days open (DO) per CM case before first artificial insemination (Santos et al. 2004) × Cost per day open (CDO) on the farm (see Annex) × Number of clinical mastitis cases.
- In cases where the average lactation curve is very flat (not common), this section may even result in a benefit, as extending the calving interval maintains production in those scenarios.

#### Subclinical mastitis

INDIRECT COSTS

Decrease in production:

The effect of SM on milk production is estimated at a loss of 0.5 kg of milk per day each time the SCC doubles starting from 50,000 cell/mL (Huijps et al. 2008). For simplification, a farm can estimate the percentage of cows in each SCC group based on the BTSCC, as previously described by den Borne et al. (2006) (see Supplementary Table S2, available as supplementary data at *Translational Animal Science* online). The final cost will be the result of multiplying the number of cows with SM in each group by the milk loss and its price.

Decreased reproductive efficiency:

Similar to CM, SM has also been reported to have an effect on reproductive performance. In this case, increases in DO up to 55 days have been described. Ultimately, the same figure used for CM was used in our economic model (20 days).

### Monte Carlo simulation and uncertainty analysis

To evaluate the impact of uncertainty in parameter estimates, a Monte Carlo simulation was performed in Microsoft Excel v. 2603 (Microsoft, Albuquerque, NM). Key parameters identified from the literature, specifically milk production losses, increased days open, probability of culling, and mortality risk, were modeled as stochastic variables. For each parameter, a normal distribution was defined based on the range of values reported in the literature (Table 2).

**Table 2:** Parameters and their corresponding range of values obtained from the literature that were used to perform a Monte Carlo simulation to calculate the cost associated with clinical and subclinical mastitis in dairy cattle herds in Northwest Spain.

Disease	Parameter	Range	References
***Clinical mastitis***	Production losses	0–9%	Hagnestam et al. (2007) Huijps et al. (2008)
	Mortality Risk Ratio	1.2–10.0	Seegers et al. (2003) Petrovski et al. (2006)
	Culling Risk Ratio	1.5–5.0	Seegers et al. (2003)
	Days Open	10–68 days	Ahmadzadeh et al. (2009) Nava-Trujillo (2019) Nava-Trujillo et al. (2010) Santos et al. (2004)
***Subclinical mastitis***	Production losses	50–100 × 10^3^ cell/mL: 0–1 kg/day 100–200 × 10^3^ cell/mL: 1.0–3.5 kg/day 200–400 × 10^3^ cell/mL: 1.5–5.0 kg/day >400 × 10^3^ cell/mL: 2.0–7.5 kg/day	Ibrahim et al. (2023) Schrick et al. (2001) Fernandes et al. (2021) Gonçalves et al. (2022)
	Days Open	0–55 days	Gonçalves et al. (2018) Reneau (1986) Huijps et al. (2008)

A total of 10,000 simulation iterations were conducted for CM and SM. In each iteration, parameter values were randomly sampled from their respective distributions and applied to the herd-level economic model, while farm-specific structural data (herd size [*n* = 100) were held constant. For farm-specific variables not derived from the literature, the observed means and standard deviations from the 63 study herds were used. The simulation generated a distribution of total costs and individual cost components. Results were summarized using quartiles (25th, 50th, and 75th percentiles) to describe variability.

In addition, a dominance analysis was conducted. For each simulation iteration, the cost component contributing the largest share of total cost was identified, and the proportion of simulations in which each component was dominant was calculated.

The Monte Carlo simulation was used to assess the robustness of cost estimates and the relative importance of cost components under parameter uncertainty, rather than to replace deterministic farm-level estimates.

### Statistical analysis

Statistical analyses were performed using IBM SPSS Statistics v. 29.0.1.0 (IBM, Armonk, NY). This study was designed as a descriptive herd-level economic assessment. Descriptive statistics were calculated for all productive, health, and economic variables. Due to the skewed distribution of several cost components, results are presented as both means (±standard deviation) and medians with 95% confidence interval (CI). Monte Carlo simulation outputs were summarized using quartiles. Differences across distributions were explored using analysis of variance (ANOVA), although interpretation focused primarily on variability and distributional patterns rather than hypothesis testing. P-values < 0.05 were considered significant.

All economic values were calculated using prices and costs corresponding to the year 2023. As all data were collected within the same annual period, no adjustment for inflation was applied. Missing data were handled through case-wise exclusion at the farm level, and only herds with complete datasets for the study period were included in the final analysis.

## Results

During 2023, data from 63 dairies were obtained. Descriptive data for farm characteristics and average costs are presented in Tables 1 and 3. The average number of milking cows per farm was 126 (22–419), with an average annual milk production per farm of 1,491,419.93 kg (151,548.00–4,980,359.70). Clinical mastitis prevalence ranged from 0.5% to 61.81%, with an average of 13.78%. Total mastitis costs (MTC) averaged €31,643.48 (2,917.71–217,799.25) per farm per year. A summary of the results from the deterministic economic analysis of the clinical and subclinical mastitis costs is presented in Table 4.

**Table 3:** Summary of the costs due to mastitis calculated from January to December 2023 in the region of study (Galicia, Northwest Spain).

	Mean	*SD*	Minimum	Maximum
***Milk price (€)***	0.51	0.02	0.46	0.55
***Vet costs (€)***	47.00	47.00	47.00	47.00
***Diagnosis costs (€)***	5.20	5.20	5.20	5.20
***Treatment costs (€)***	45.35	15.36	7.40	89.15
***Treatment duration (Days)***	8.39	1.19	4.00	12.50
***Employee salary (€)***	1467.96	156.63	1323.00	1834.00
***Employee working hours per week***	40.00	40.00	40.00	0.00
***Cull cow price (€)***	671.89	171.77	494.95	1150.56
***Replace cow Price (€)***	1734.92	107.63	1650.00	2100.00

**Table 4:** Median, 95% confidence interval (CI), mean, standard deviation (*SD*), maximum and minimum from clinical (CM) and subclinical (SM) mastitis costs (€) per farm and year according to the deterministic economic analysis performed for 63 cattle dairy farms in Northwest Spain.

	Median	95% CI	Mean	*SD*	Minimum	Maximum	Cost/cow^a^
***CM Diagnostic costs***	518.72	484.86–995.15	740.00	1013.09	56.12	7606.69	5.86
***CM Treatment costs***	382.01	457.36–770.80	614.08	622.29	35.80	3016.45	4.86
***CM Non-saleable milk costs***	1414.59	1349.22–3152.46	2250.84	3580.04	70.19	27,244.00	17.81
***CM Death costs***	88.26	128.62–400.55	264.58	539.88	0.00	3867.10	2.09
***CM Labor costs***	1186.81	1086.11–2427.34	1756.72	2662.79	116.00	20,364.53	13.90
***CM Direct costs***	3527.09	3550.33–7702.13	5626.23	8242.72	278.11	62,098.77	44.52
***CM Losses in milk production***	2206.92	2179.87–5179.33	3679.60	5954.92	138.03	45,663.40	29.12
***CM Culling rate***	2477.57	2340.28–3728.53	3034.41	2756.16	115.66	16,091.68	24.01
***CM Days open***	521.28	560.61–1084.89	2836.80	1040.86	57.68	7579.78	22.45
***CM Indirect costs***	5155.84	5152.68–9920.83	7536.76	9466.37	315.05	69,334.85	59.64
***CM Total costs (CMTC)***	8768.25	8712.64–17,613.33	13,162.99	17,670.86	593.16	131,433.62	104.16
***SM Losses in milk production***	10,855.60	11,917.07–19,370.32	15,643.70	14,797.20	626.04	72,781.45	123.79
***SM Days open***	1246.83	1603.02–4070.58	2836.80	4898.92	0.00	25,625.54	22.45
***SM Total costs (SMTC)***	12,199.35	13,664.79–23,296.21	18,480.56	19,121.59	626.04	86,365.63	146.24
***Mastitis total costs (MTC)***	24,153.98	23,501.37–39,785.60	31,643.48	32,329.65	2917.71	217,799.25	250.40

a This value was calculated by dividing the mean cost by 126.37 (mean number of cows per farm).

### Clinical mastitis

**Figure 1:**
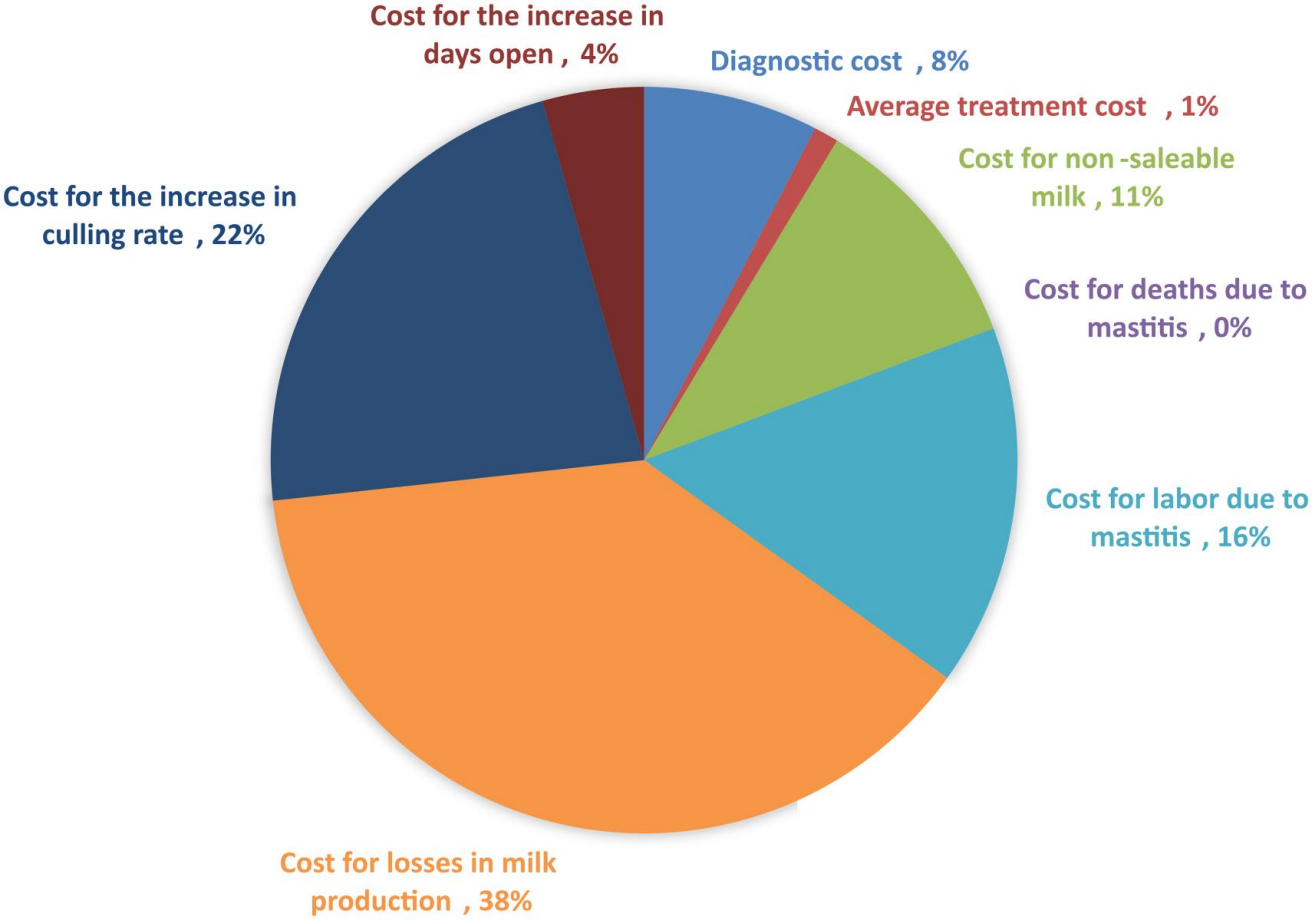
Distribution of the costs due to clinical mastitis in 63 dairy cattle herds located in Northwest Spain.

**Figure 2:**
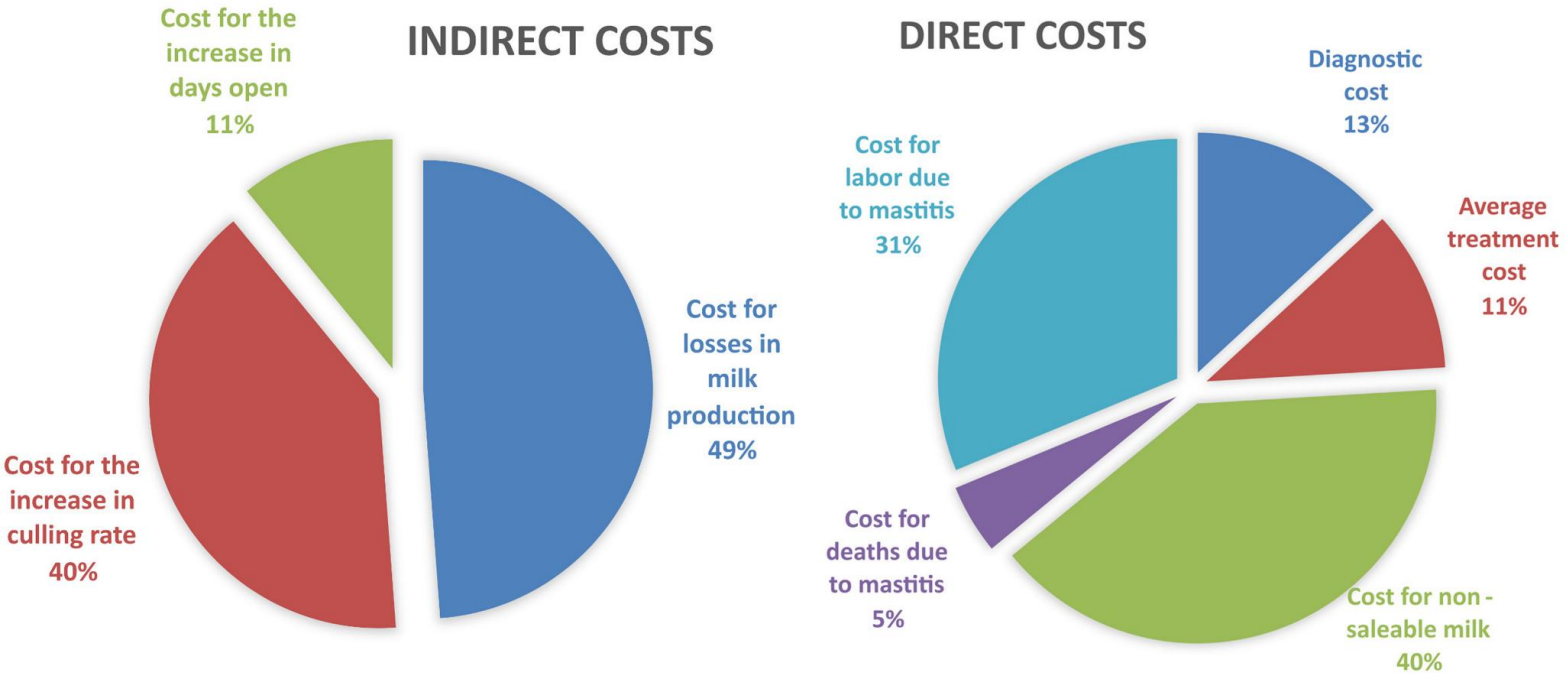
Distribution of the indirect and direct costs due to clinical mastitis in 63 dairy cattle herds located in Northwest Spain.

As can be seen in Fig. 1, total costs associated with CM (CMTC) were distributed between indirect (57.26%) and direct (42.74%) costs. The distribution of the individual direct and indirect cost components is presented in Fig. 2. The largest indirect cost component was milk production losses, accounting for 38% of CMTC. This corresponded to an average annual loss of 7214.90 kg of milk, with an associated cost of €3679.60 (138.03–45,663.40). Increased culling rate was the second most important indirect cost of CM (€3034.41; 115.66–16,091.68), followed by reproductive losses associated with increased days open (€2836.80; 0–25,625.54). Altogether, total indirect costs reached €7536.76 (315.05–69,334.85).

Among direct costs, discarded milk represented the largest component (40%), with an average cost of €2250.84 (70.19–27,244.00). Labor costs due to mastitis were the second most important component (31%), achieving €1756.72 (116.00–20,364.53), followed by diagnostic cost (€740.00; 56.12–7606.69), and treatment cost (€614.08; 35.80–3016.45). Overall, CMTC averaged €13,162.99 per farm per year (593.16–131,433.62).

### Subclinical mastitis

Total costs associated with SM (SMTC) averaged €18,480.56 per farm per year (626.04–86,365.63) (Table 6). Milk production losses accounted for the majority of SMTC (84.65%), corresponding to €15,643.70 (626.04–72,781.45). Reproductive losses due to increased days open represented the remaining 15.35% with an average cost of €2836.80 (0.00–25,625.54).

### Uncertainty analysis (Monte Carlo simulation)

A Monte Carlo simulation (10,000 iterations) was performed to assess the impact of parameter uncertainty on cost estimates. The simulation revealed substantial variability in total mastitis costs and their individual components (Table 5). Median total cost was €62,787, with a wide interquartile range, reflecting the sensitivity of the model to parameter variation. Among cost components, culling showed the highest absolute values for CM costs in the 1st, 2nd and 3rd quartiles, while increase in days open represented the largest component in the 4th quartile. Regarding SM, increase in days open had the highest absolute values in the 4th quartile, while losses in milk production were predominant in the 1st. 2nd and 3rd quartile.

**Table 5:** Summary of the mastitis costs in cattle dairy herds resulting from the Monte Carlo simulation (*n* = 10,000) using the parameters and range values obtained from the literature.

		1st Quartile			2nd Quartile			3rd Quartile			4th Quartile		
		Mean	*SD*	%	Mean	*SD*	%	Mean	*SD*	%	Mean	*SD*	%
**Clinical mastitis (CM)**	Diagnostic cost	684.43	544.63	−0.25	607.53	514.82	0.66	785.12	536.71	0.61	973.14	578.74	0.16
	Average treatment cost	595.42	530.85	−0.22	530.88	511.39	0.58	679.83	539.83	0.53	841.51	593.95	0.14
	Cost for non-saleable milk	1900.91	1615.25	−0.70	1681.00	1532.79	1.82	2188.72	1621.52	1.71	2735.08	1779.51	0.44
	Cost for deaths due to mastitis	3324.06	2987.62	−1.22	3563.55	3120.59	3.86	3767.05	3170.03	2.94	3793.93	3280.87	0.62
	Cost for labor due to mastitis	83.12	67.08	−0.03	73.70	63.19	0.08	95.23	66.31	0.07	118.17	71.82	0.02
	**Total direct costs CM**	**6587.94**	**4084.20**		**6456.66**	**3988.22**		**7515.95**	**4082.87**		**8461.84**	**4407.20**	
	Cost for losses in milk production	2933.22	3353.15	−1.08	2661.12	3165.74	2.88	3331.30	3310.30	2.60	3788.94	3490.78	0.62
	Cost for the increase in culling rate	44,754.81	18,076.88	−16.42	50,445.08	14,858.01	54.65	57,879.87	17,870.12	45.23	58,217.09	19,109.28	9.45
	Cost for the increase in days open	−13,0221.48	1,185,392.38	47.79	3224.63	9323.71	3.49	15,039.77	15,343.41	11.75	20,9319.57	1,717,524.79	33.99
	**Total indirect costs CM**	**−82,533.45**	**118,4196.02**		**56,330.83**	**12,709.49**		**76,250.94**	**17,268.91**		**2,71,325.59**	**1,716,824.86**	
	**TOTAL CM**	**−75,945.51**	**1,183,999.90**		**62,787.49**	**13,130.24**		**83,766.90**	**18,498.94**		**279,787.43**	**1,716,799.21**	
**Subclinical mastitis (SM)**	Cost for losses in milk production	22,441.43	7620.57	−8.24	24,998.13	8495.89	27.08	27,182.72	8855.85	21.24	27,297.98	9359.82	4.43
	Cost for the increase in days open	−21,9001.75	3,275,030.26	80.37	4524.92	10,492.83	4.90	17,023.31	16,242.61	13.30	308,709.85	5,189,253.39	50.13
	**TOTAL SM**	**−196,560.32**	**327,5096.10**		**29,523.05**	**12,446.78**		**44,206.03**	**18,120.12**		**336,007.83**	**518,9170.25**	
	**TOTAL MASTITIS**	**−27,2505.83**	**4,215,738.06**		**92,310.54**	**9212.41**		**127,972.93**	**12,956.02**		**615,795.26**	**6,660,894.12**	

Bold values indicate total cost categories (total direct costs, total indirect costs, total clinical mastitis costs, total subclinical mastitis costs, and total mastitis costs).

**Table 6:** Mean, standard deviation (*SD*), maximum, and minimum of the relative total costs (€) of clinical (CMTC) and subclinical (SMTC) mastitis, and the total mastitis cost (MTC) per case/cow and year, according to the deterministic economic analysis performed for 63 dairy cattle farms in the northwest of Spain.

	Mean	*SD*	Minimum	Maximum
***CMTC per case***	920.40	144.50	541.66	1301.51
***CMTC per cow***	122.45	101.85	5.63	522.28
***SMTC per case***	939.87	714.04	249.04	4210.33
***SMTC per cow***	138.78	64.84	16.92	360.65
***MTC per cow***	261.23	129.52	61.26	668.10

### Dominance of cost components

Dominance analysis revealed that culling-related costs (eliminated animals) were the most frequent primary driver of total economic losses, accounting for 72.2% of simulations. Costs associated with increased days open were dominant in 21.9% of cases. In contrast, milk-related losses were rarely the largest contributor, representing 5.9% of simulations.

### Relative costs

When the costs are distributed among the total amount of cows, as can be seen in Table 6, MTC had a cost of €261.23 per cow (61.26–668.10). When mastitis types were analyzed separately, CMTC was €920.40 per case (541.66–1301.51) and €122.45 per cow (5.63–522.28), while SMTC was €939.87 per case (249.04–4210.33) and SMTC was €138.78 per cow (16.92–360.65).

## Discussion

In the present study, a comprehensive herd-level economic model was applied using real farm data from Northwest Spain, integrating both direct and indirect cost components derived from the literature.

The prevalence of mastitis observed in the current study (13.78%) was lower than that reported in other studies conducted in Spain (24.76%) and internationally (∼19%) (Pérez-Cabal et al. 2008; Aghamohammadi et al. 2018; Borş et al. 2023). On the other hand, our rate of CM is higher than other studies, which reported an incidence of 3% (Corrêa et al. 2024) and 6% (Rollin et al. 2015). These variability across studies could be explained by the differences in herd characteristics, management practices, and case definitions, especially considering the onset time of the disease, as cattle < 150 days in milk (DIM) had 2 times less chances to have mastitis than cattle > 305 DIM (Schunig et al. 2024).

Our economic model showed that milk production losses appeared to be the largest contributor to the CMTC, followed closely by culling costs, which is consistent with several previous studies (Halasa et al. 2007; Heikkilä et al., 2012; Borş et al. 2023; Corrêa et al. 2024). However, when parameter uncertainty was incorporated through Monte Carlo simulation, a different pattern was observed. The dominance analysis revealed that culling-related costs were the most frequently primary contributor to total economic losses, accounting for 72.3% of simulated scenarios, followed by reproductive losses associated with increased days open (21.9%). The disagreement between the deterministic model and the simulation can be explained by the range variability among the parameters included in the analysis and the differences in the herd size considered. In this regard, milk losses might represent the most stable and consistently observable economic consequence of mastitis across farms, whereas culling and reproductive losses may be highly sensitive to variations in herd management, milk production, replacement policies, reproductive performance, and disease severity (culling risk ratio varying from 1.5 to 5.0; days open ranging from 10–68 days in CM and 0–55 days in SM). This is illustrated by the negative values observed in the 1st quartile due to days open that could have resulted from the combination of increased days open in cows with high production and a flat lactation curve. Negative values observed in some Monte Carlo iterations reflected theoretical scenarios in which extended lactation persistence partially compensated for reproductive losses under flat lactation curves. These values should not be interpreted as a true economic benefit of mastitis, but rather as a consequence of the probabilistic structure of the model. Additionally, it should be considered that the simulation was performed for a model farm of 100 cows, while the economic model included the variable size of the farms enrolled in the study. Consequently, this difference between analyses should be interpreted with caution and economic models should be customized according to the characteristics of each farm.

Several studies have identified culling-related costs as one of the main economic consequences of CM. Aghamohammadi et al. (2018) reported culling as the largest contributor to CM-associated losses, whereas other studies have ranked culling as the second most important factor (Halasa et al. 2007; Corrêa et al. 2024). These variations could indicate that the relative contribution of culling to the CMTC may vary considerably depending on the production system, herd characteristics, and methodological approach used. In the present study, 6.01% of culled cows were attributed to CM, which was slightly lower than the 11.1% reported by Corrêa et al. (2024). Halasa et al. (2007) concluded that culling was the second most important cost due to mastitis, but also highlighted the difficulty of accurately estimating the costs due to the multiple factors influencing culling decisions. In contrast, some studies identified treatment and veterinary expenses as the second largest CM-related cost component (Pérez-Cabal et al. 2008; Heikkilä et al., 2012; Borş et al. 2023), likely reflecting differences in mastitis incidence (24.76% vs 13.78%), herd characteristics, and veterinary service costs (€180 vs €97) compared to our study. Similarly, discarded milk and labor costs were generally reported as secondary contributors to total CM costs (Heikkilä et al., 2012; Aghamohammadi et al. 2018). Differences between studies may be explained by variation in labor valuation (2 vs 1 hour), treatment protocols, milk price, and herd management practices. In the present study, these components represented a comparatively smaller proportion of the total economic burden than culling-related and reproductive costs.

According to the authors’ knowledge, the distribution of SM costs has only been analyzed in one study conducted in 2018, which concluded that milk yield reduction and culling cost represented the 97% of economic losses associated with SM (Aghamohammadi et al. 2018). In the present study, only milk production losses and costs due to increased days open were attributed to SM. Similar to the findings reported by Aghamohammadi et al. (2018), the largest cost component in our study was the decrease in milk production representing 84.65% of total SM costs. However, reproductive parameters were not included in their economic model, which were categorized as the most important contributor by the Monte Carlo simulation for the 25% of farms with greater total mastitis costs. Conversely, culling costs were not incorporated into the SM model because, as previously indicated by Aghamohammadi et al. (2018), culling attributable specifically to CM and SM is rarely reported separately in the literature. Other authors have evaluated SM-related economic losses through different approaches. Dillon et al. (2015) estimated the economic impact of SM based on reductions in gross margin associated with increased bulk tank somatic cell count (SCC), whereas Hogeveen et al. (2011) estimated milk production losses associated with SM at €13 per cow when milk price was €0.12/kg.

Considering relative costs, MTC reached €261.23 per cow (61.26–668.10), which is substantially higher than the €28 per cow reported in the review by Halasa et al. (2007). Similarly, the CMTC observed in our study (€920.40 per case [541.66–1,301.51]) exceeded the €266 per case described in the same review, although those authors also reported considerable variability among studies. Likewise, Pérez-Cabal et al. (2008) estimated economic losses of €29 per cow and €73.93 per CM case in Spanish dairy farms, values notably lower than those observed in the present study. These differences may be partially explained by the lower milk price reported in their study (€0.31/kg) compared with the average milk price during the present study period (€0.51/kg). In addition, increases in production costs and milk prices over recent decades (between 21% and 34%) may contribute to the higher economic estimates observed in more recent studies (Bełdycka-Bórawska et al. 2021).

Contemporary research has generally reported higher CMTC than older reviews, although still lower than those observed in the present study (Heikkilä et al., 2012; Rollin et al. 2015). Rollin et al. (2015) estimated a CMTC of $444 per case, approximately half the value obtained in our study. Additionally, Heikkilä et al. (2012) reported CM costs of €155 and €191 per cow in Ayrshire and Holstein cattle, respectively, which were higher than the €122.45 per cow observed in the present study. However, their estimated cost per CM case (€822 for Ayrshire and €848 for Holstein cows) remained slightly lower than the values obtained in our analysis. Finally, recent studies conducted in Romania and the United States also reported lower CMTC per case than those observed in the present work (Borş et al. 2023; Rodriguez et al. 2024). Borş et al. (2023) estimated a relative cost of $153.39 per CM case in a single Romanian dairy farm, whereas Rodriguez et al. (2024) reported a CM cost of $521 per case. It should be noted that the different results observed among studies might be explained, mainly, by differences in milk price between the regions of study, and the production level of the animals enrolled, as milk losses turned out to be an important contributor to mastitis cost. Moreover, the variations reported in herd size and structure may also have an important influence in the heterogeneous results, especially regarding relative costs. Lastly, variations in the parameters included in the economic model might also partially explain these discrepancies.

As for SM, relative costs were also evaluated by Aghamohammadi et al. (2018), who reported a cost of €348.59 per dairy cow, higher than €138.78 (16.92–360.65) per cow observed in the present study. This difference may be partially explained by the inclusion of culling costs in their analysis. Similarly, Rodriguez et al. (2024) estimated SM costs at $767 per cow in cases occurring during early lactation, whereas the present study did not differentiate costs according to stage of lactation. Greater milk losses due to SM are expected in early lactation, close to the peak, while the combination of the different periods along lactation could have substantially diluted this effect in the cases included in our study.

Overall, these results have broader implications beyond the specific regional context. The herds enrolled in the study followed production systems and management practices comparable to those of dairy cattle farms located in the Atlantic European regions, characterized by intensive production systems, high milk yield, and similar climatic conditions. Therefore, the economic patterns observed in the present study may be applicable to dairy herds operating under comparable regimes. From a practical perspective, the present study provides veterinarians, producers, and herd advisors with a structured framework for estimating mastitis-associated losses using routine available farm data. This approach might be of help to identify the major economic drivers within individual herds and support decision-making regarding prevention, monitoring, and udder health strategies.

## Conclusion

Mastitis represented a substantial economic burden in the dairy farms enrolled in the present study, with an average total mastitis cost of €31,643.48 per farm per year. Although the deterministic economic analysis identified milk production losses as the main contributor to mastitis-associated costs in both clinical and subclinical mastitis, the uncertainty analysis demonstrated that culling-related costs and reproductive losses associated with increased days open became the dominant economic drivers in a substantial proportion of simulated scenarios. Therefore, economic models evaluating mastitis-associated losses should be carefully adapted to individual herd characteristics, as variations in herd size and management, milk production, and reproductive performance may substantially influence the final economic estimates. These results emphasize the importance of implementing preventive and monitoring strategies aimed not only at reducing mastitis prevalence, but also at minimizing long-term reproductive impairment and premature culling in dairy herds. Furthermore, the present study provides a structured herd-level economic framework based on real farm data that may be applicable to dairy farms operating under similar Atlantic European production systems. Further steps should focus on the influence of pathogen-specific effects, lactation stage, and the impact of management strategies on mastitis-associated costs, as well as refining probabilistic economic models to improve precision and applicability of herd-level economic assessments.

### Limitations

Farms were voluntarily enrolled in the study. Therefore, potential selection bias should be acknowledged. Additionally, all participating herds were voluntarily enrolled in a milk quality and recording program, which may limit extrapolation of the results to dairy farms that do not routinely participate in structured monitoring systems. In addition, the economic model relies on published coefficients for certain biological effects of mastitis. Although these parameters were selected following a comprehensive literature review, their magnitude may vary across production systems and management conditions. To address this source of uncertainty, a Monte Carlo simulation was performed, demonstrating that the relative contribution of individual cost components may change substantially depending on the assumptions applied. Finally, preventive costs associated with mastitis control (e.g., teat disinfection and routine veterinary monitoring, among others) were not included in the analysis and should be considered when interpreting the total economic burden of mastitis. Despite these limitations, the use of real farm-level production and economic data, together with a standardized herd-level economic model, provides a robust and practical framework for estimating mastitis costs under intensive dairy farming conditions.

## List of Abbreviations

CM: Clinical Mastitis
SM: Subclinical Mastitis
MTC: Mastitis Total Costs
CMT: California Mastitis Test
CI: Confidence Interval
CMTC: Clinical Mastitis Total Costs
SMTC: Subclinical Mastitis Total Costs
BTSCC: Bulk Tank Somatic Cells Count
SCC: Somatic Cell Count
DO: Days Open
CDO: Cost of Days Open
DIM: Days In Milk
SD: Standard Deviation
OR: Odds Ratio
IGE: Galician Institute of Statistics (Instituto Gallego de Estadística)
